# An immunofluorescence assay for extracellular matrix components highlights the role of epithelial cells in producing a stable, fibrillar extracellular matrix

**DOI:** 10.1242/bio.025866

**Published:** 2017-10-15

**Authors:** Omar S. Qureshi, Hélène Bon, Breda Twomey, Gill Holdsworth, Kirsty Ford, Marianne Bergin, Linghong Huang, Mariusz Muzylak, Louise J. Healy, Vanessa Hurdowar, Timothy S. Johnson

**Affiliations:** UCB Pharma, Berkshire SL1 3WE, UK

**Keywords:** Collagen, Epithelial cell, Fibroblast, Extracellular matrix, Fibrosis, Fibronectin, Kidney, Transforming growth factor beta (TGF-β), Transglutaminase

## Abstract

Activated fibroblasts are considered major drivers of fibrotic disease progression through the production of excessive extracellular matrix (ECM) in response to signals from damaged epithelial and inflammatory cells. Nevertheless, epithelial cells are capable of expressing components of the ECM, cross-linking enzymes that increase its stability and are sensitive to factors involved in the early stages of fibrosis. We therefore wanted to test the hypothesis that epithelial cells can deposit ECM in response to stimulation in a comparable manner to fibroblasts. We performed immunofluorescence analysis of components of stable, mature extracellular matrix produced by primary human renal proximal tubular epithelial cells and renal fibroblasts in response to cytokine stimulation. Whilst fibroblasts produced a higher basal level of extracellular matrix components, epithelial cells were able to deposit significant levels of fibronectin, collagen I, III and IV in response to cytokine stimulation. In response to hypoxia, epithelial cells showed an increase in collagen IV deposition but not in response to the acute stress stimuli aristolochic acid or hydrogen peroxide. When epithelial cells were in co-culture with fibroblasts we observed significant increases in the level of matrix deposition which could be reduced by transforming growth factor beta (TGF-β) blockade. Our results highlight the role of epithelial cells acting as efficient producers of stable extracellular matrix which could contribute to renal tubule thickening in fibrosis.

## Abstract (precis)

**Summary:** Using an immunofluorescence extracellular matrix, we show that epithelial cells can deposit extracellular matrix components in a comparable manner to fibroblasts highlighting their role as potential targets in fibrotic disease.

## INTRODUCTION

Chronic kidney disease (CKD) is a significant public health burden with a prevalence exceeding 10% in the adult population (Mills et al., 2015). CKD is characterised by a reduction in glomerular filtration rate and increased urine albumin excretion caused by damage to the kidney (Eckardt et al., 2013). This can lead to end-stage renal failure and may also be associated with co-morbidities through broader effects on body homeostasis. CKD shares patho-biology with other fibrotic diseases and represents a major challenge in global health.

The progressive deposition and accumulation of excess extracellular matrix (ECM) is a defining feature of all fibrotic diseases. In the kidney, following nephron injury, the wound response leads to the production of ECM during the repair process (Duffield, 2014). In the case of chronic kidney disease, this repair process fails to resolve and the continuing accumulation of matrix leads to progressive fibrosis and scarring. The accumulation of ECM and kidney fibrosis correlates closely with the decline in kidney function and are consistent features of end-stage CKD (Genovese et al., 2014).

Kidney fibroblasts and myofibroblasts are believed to be the major effector cells that both synthesise and deposit fibrillary ECM components in renal interstitial fibrosis (Strutz and Zeisberg, 2006). These cells can be activated by cytokines including transforming growth factor beta (TGF-β) (Strutz et al., 2001; Zeisberg et al., 2000; Ignotz and Massague, 1986) and tumour necrosis factor alpha (TNFα) (Guo et al., 2001; Stenvinkel et al., 2005) as well as stress stimuli including hypoxia (Norman et al., 2000). These factors can lead to increased production of matrix components including fibronectin, collagen I and collagen III.

The role of the epithelial cell in matrix deposition during fibrosis is less well established, although they can clearly respond to fibrotic stimuli and produce components of the extracellular matrix (Orphanides et al., 1997; Ng et al., 1998; Humphreys et al., 2010). Regardless of whether these tubular epithelial cells undergo full conversion to a myofibroblast phenotype, they are positioned such that they could make a significant contribution to tubular thickening and dysfunction if they were to deposit significant amounts of stable ECM (Louis and Hertig, 2015). An additional mechanism through which epithelial cells can contribute to the progressive accumulation of extracellular matrix is through the production of enzymes such as TG2 and LOXL enzymes. These can cross-link components of the extracellular matrix to increase matrix stability (Gross et al., 2003; Kleman et al., 1995) as well as increasing its resistance to degradation (Johnson et al., 1999).

Previous studies have typically examined the ability of pro-fibrogenic cytokines to increase ECM synthesis as well as the changes in the relative production of ECM proteins (Forino et al., 2006). These studies have been influential in building our understanding of pro-fibrogenic cytokines and growth factors. However they did not necessarily establish an effect on mature ECM. Studies in rodent cell lines again highlight this production but do not address the relative capacity of epithelial cells compared to fibroblasts in human cells (Creely et al., 1992). The latter is important as epithelial cell expression of matrix components has been reported in diabetic nephropathy (Razzaque et al., 1995), and we frequently observe in our animal models of tubulointerstitial fibrosis the expansion of the ECM before clear cellular infiltration (Fig. S1). We have therefore developed an immunofluorescence assay using human epithelial cells where we strip the cellular components to allow us to focus directly on the remaining deposited ECM. Using this assay we have sought to test the hypothesis that primary human renal proximal tubular epithelial cells can deposit an ECM of comparable quality and quantity to primary renal fibroblasts, and that this is brought about in part by fibroblast-epithelial cell crosstalk.

## RESULTS AND DISCUSSION

### Characterisation of primary epithelial cells and fibroblasts

**Fig. 1.**
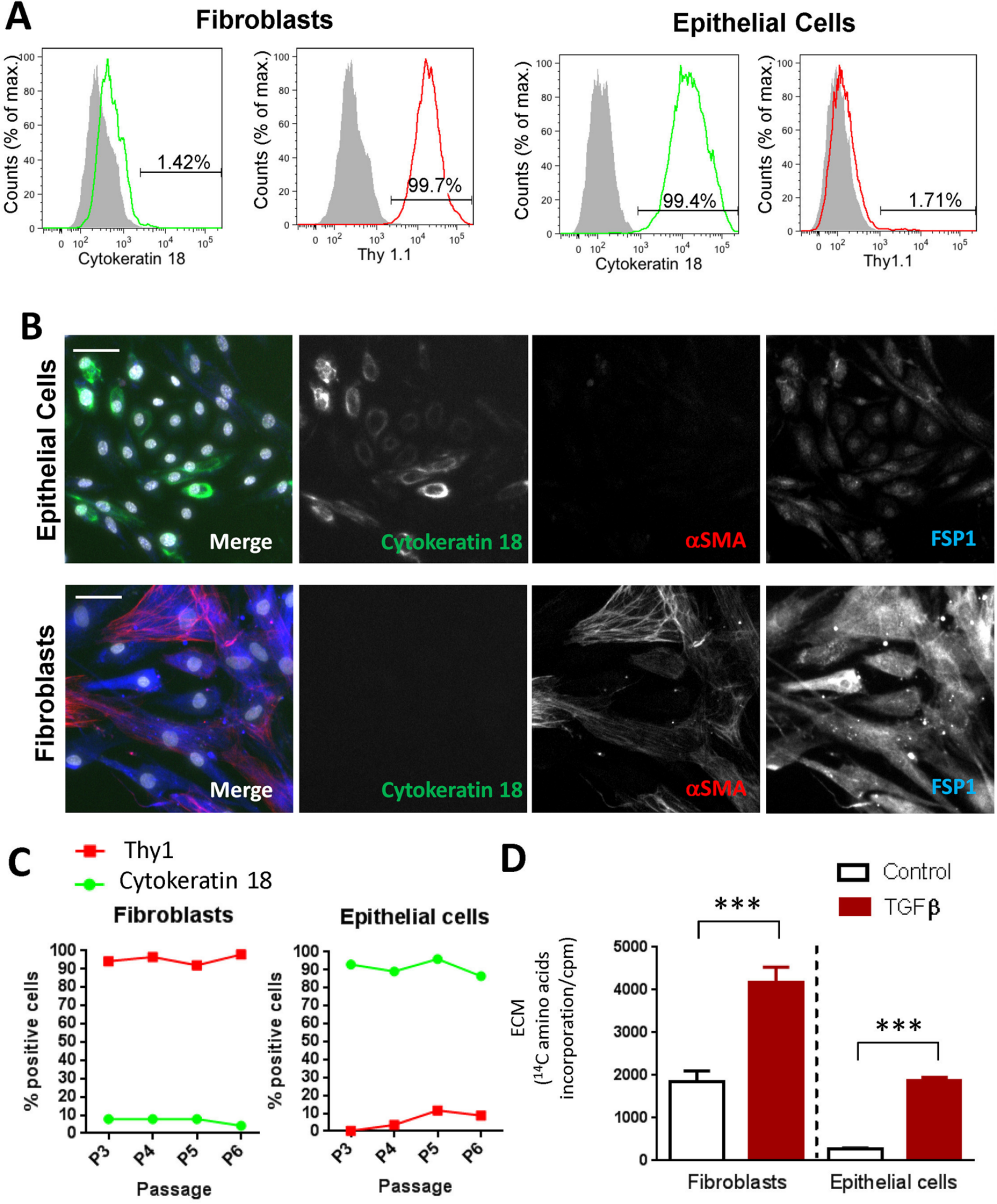
**Phenotypic characterisation of primary human tubular epithelial cells and human renal fibroblasts.** (A) Flow cytometric analysis of cytokeratin18 and Thy1.1 staining in primary human epithelial cells and fibroblasts. Grey shading indicates unstained cells. (B) Immunofluorescence staining of fibroblasts and epithelial cells for FSP1 (blue), α-smooth muscle actin (red) and cytokeratin 18 (green). Scale bars: 50 µm. (C) Flow cytometric analysis of cytokeratin and Thy1 expression on fibroblasts and epithelial cells over increasing passage number. (D) Accumulation of total extracellular matrix produced by fibroblasts and epithelial cells cultured for 6 days with or without stimulation using 10 ng/ml TGFβ1 in the presence of ^14^C amino acids. Cells were stripped and extracellular matrix accumulation was measured by scintillation counting. Graph shows data from five independent experiments with at least four replicates per experiment, statistical analysis performed on means from independent experiments, error bars indicate mean±s.e.m.; ***P<0.001, paired *t*-test performed on means from independent experiments.

In order to study the deposition of matrix from human renal proximal tubular epithelial cells (RPTECs) and human renal fibroblasts (HRFs) we wanted to perform analysis on human cells that were as close to their primary sources as possible for optimal translational relevance. We purchased primary isolations of these cell types and performed an initial characterisation. As expected, the epithelial cells showed high expression of cytokeratin 18, and were Thy1.1 negative (Fig. 1A,B). In addition, these cells were α-smooth muscle actin negative and showed low to undetectable levels of Fsp-1. In contrast the fibroblasts were negative for cytokeratin 18 staining, showed strong staining for Thy1.1 and FSP1, and approximately 30% were α-smooth muscle actin positive (Fig. 1B). These phenotypes remained stable up to six passages (Fig. 1C) and cells were used for subsequent assays up to this point. This provided us with primary cultures of human renal cells in which we could investigate the accumulation of mature ECM.

### Comparable deposition of fibrillar extracellular matrix by epithelial cells and fibroblasts

Although fibroblasts are generally considered the primary cell contributing to the mature ECM, tubular epithelial cells express several components of the extracellular matrix including fibronectin and collagen IV (Bürger et al., 1998; Razzaque et al., 1995). Importantly, these cells also have the capacity to express the fibrillar collagens I and III which are associated with renal disease. We therefore wanted to study the accumulated matrix that was stably deposited by each cell type.

Initially, we performed a comparison of the total extracellular matrix produced by epithelial cells and fibroblasts using an established radioactive ^14^C amino acid incorporation assay to measure total deposited extracellular matrix. As expected, fibroblasts produced significant amounts of matrix under basal conditions and this was approximately doubled by treatment with TGF-β1 (Fig. 1D). Whilst the ECM deposited by epithelial cells under basal conditions was approximately fourfold lower than HRFs, this was considerably increased in response to stimulation (three- to fivefold) reaching the basal level in fibroblasts.

**Fig. 2.**
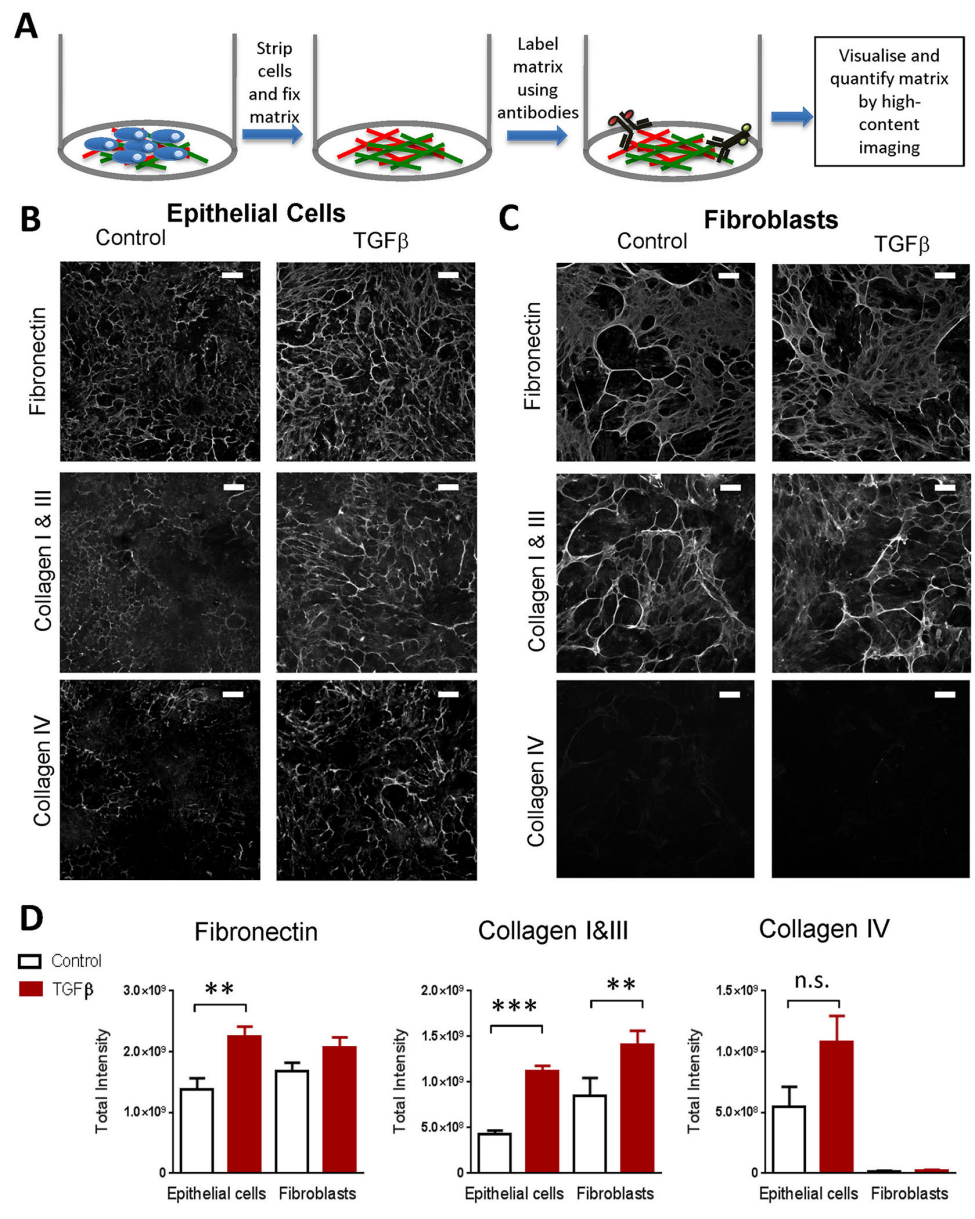
**Stimulated proximal tubular epithelial cells produce a mature, deposited extracellular matrix comparable to that generated by fibroblasts.** (A) Diagram showing the method for immunofluorescence analysis of deposited ECM. (B) Epithelial cells cultured for 6 days with or without stimulation with 10 ng/ml TGFβ1. Cells were then stripped, extracellular matrix fixed, and cells stained using antibodies against collagen I, III, IV and fibronectin. Each panel shows an image of one representative field from four independent experiments. Scale bars: 100 µm. (C) Fibroblasts cultured with or without stimulation for 6 days and stained as in B. Scale bars: 100 µm. (D) The graphs show the results of the quantification of fibrillary extracellular matrix from images as in B and C. Results are the mean from four independent experiments with 6 replicates per experiment, *t*-test performed on means from independent experiments, **P<0.01, ***P<0.001; error bars indicate mean±s.e.m.

To understand in more detail the components and organisation of this mature deposited extracellular matrix, we used an imaging-based immunofluorescence approach to visualise key constituents of the mature extracellular matrix. This allowed us to investigate the fibrillar collagens that are associated with the fibrotic matrix. To do this, we first stripped away the cultured cells, fixed the deposited extracellular matrix, labelled matrix components using antibodies, then visualised and quantified this matrix by high-content imaging (Fig. 2A, and see Materials and Methods). Under basal conditions we observed comparable deposition of fibronectin by fibroblasts and epithelial cells (Fig. 2B-D). Basal deposition of collagen I and III obtained with fibroblasts was approximately two- to threefold higher than the levels obtained using epithelial cells. In contrast, epithelial cells readily deposited collagen IV but fibroblasts did not. Following stimulation with TGF-β1, epithelial cells showed increases in the levels of all three ECM components which accumulated in a fibrillar matrix structure and at levels not significantly different to fibroblasts (Fig. 2B-D). Qualitatively, the fibroblast matrix appeared to have thicker collagen fibrils with areas that were sparse. In epithelial cells, the matrix was more even and composed of slightly thinner fibrils. Epithelial cells were therefore able to deposit an equivalent level of the fibrosis-associated matrix components to fibroblasts.

**Fig. 3.**
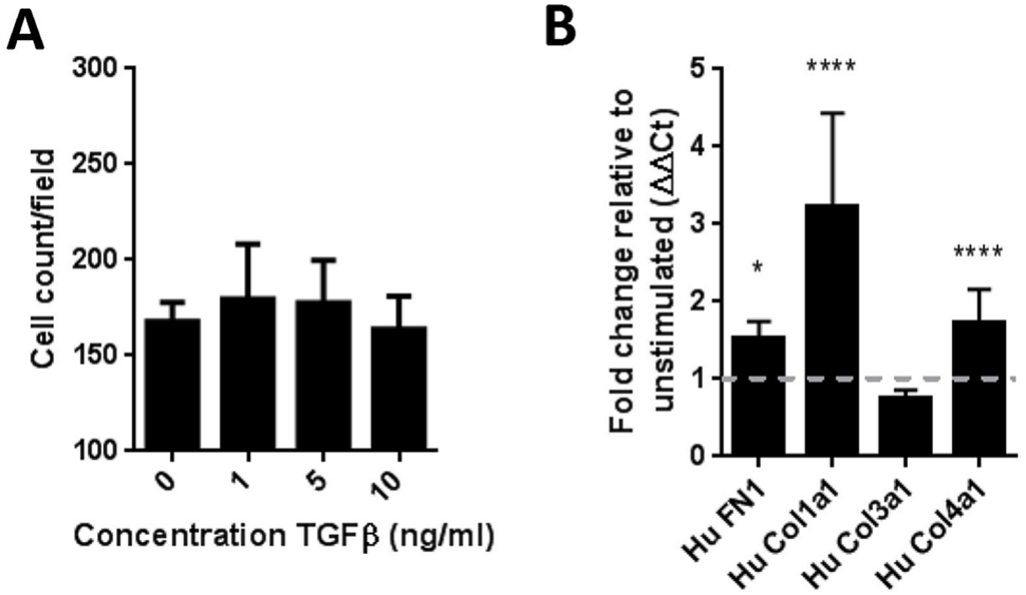
**Primary human renal proximal tubular epithelial cells activated by TGFβ1 have enhanced extracellular matrix production.** (A) No change in cell counts of epithelial cells on stimulation with 10 ng/ml TGFβ1. After 6 days, cells were fixed and nuclei were stained with DAPI and images analysed for cell count per field. One representative experiment of three shown. Data shows mean±s.d. for three replicate wells. (B) mRNA for extracellular matrix component transcripts was determined by qRT-PCR after 48 h in culture with TGFβ1. Data shows mean±s.d. for four independent experiments. One-way ANOVA performed versus unstimulated control, ****P<0.001; *P<0.05.

To understand if proliferation was responsible for this increased deposition of extracellular matrix we initially performed cell counts of epithelial cells following stimulation with TGF-β1. Whilst we did not observe a significant increase in epithelial cell numbers (Fig. 3A), we observed increased expression of mRNA for the matrix components fibronectin, collagen I and collagen IV (Fig. 3B).

Taken together, these results show that epithelial cells are capable of producing a stable, fibrillar matrix with fibrosis-associated components in quantities comparable to fibroblasts in response to cytokine stimulation.

### Sensitivity of epithelial cells to stress stimuli, hypoxia and co-culture

*In vivo*, the position of epithelial cells leaves them exposed to extracellular stresses which could trigger the onset of fibrosis. Chronic exposure to hypoxia (Fine and Norman, 2008) or chemical stresses (Debelle et al., 2002) *in vivo* lead to extracellular matrix deposition, fibrosis and chronic kidney disease.

**Fig. 4.**
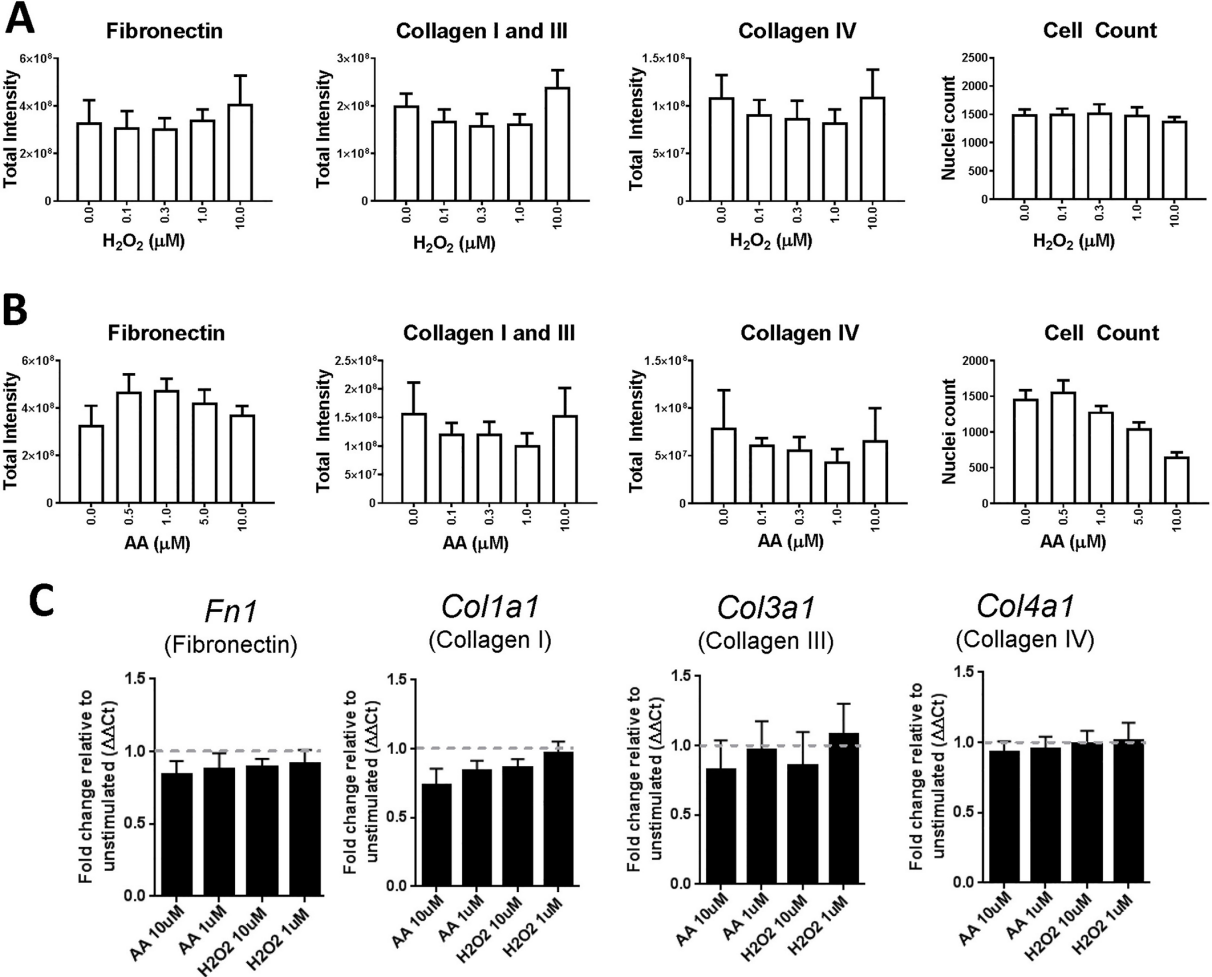
**Hydrogen peroxide and aristolochic acid have no effect on extracellular matrix deposition in RPTECs.** (A) Effect of H_2_O_2_ on extracellular matrix deposition as assayed using the immunofluorescence ECM assay. Epithelial cells were incubated for 6 days with indicated concentrations of H_2_O_2_ (white-filled bars). (B) As A, except aristolochic acid (AA) was used as the stimulus. Representative example from two independent experiments is shown. (C) mRNA for extracellular matrix component transcripts determined by qRT-PCR after 48 h in culture with AA or H_2_O_2_. Data shows mean±s.d. for four independent experiments.

We therefore investigated the ability of two stress stimuli to induce matrix deposition in isolated epithelial cells: H_2_O_2_ to generate oxidative stress (Okamura and Pennathur, 2015), and aristolochic acid to mimic kidney injury (Luciano and Perazella, 2015). In our isolated system, these triggers did not cause a significant increase in the stable deposition of fibronectin or collagen at concentrations that did not significantly affect cell number (Fig. 4A,B). Consistent with this, we did not see an increase in mRNA for fibronectin, collagen I, collagen III or collagen IV (Fig. 4C). This lack of induction may reflect the need for additional cell types such as immune cells to modulate the response, or the requirement for a more chronic stimulus to induce extracellular matrix deposition.

**Fig. 5.**
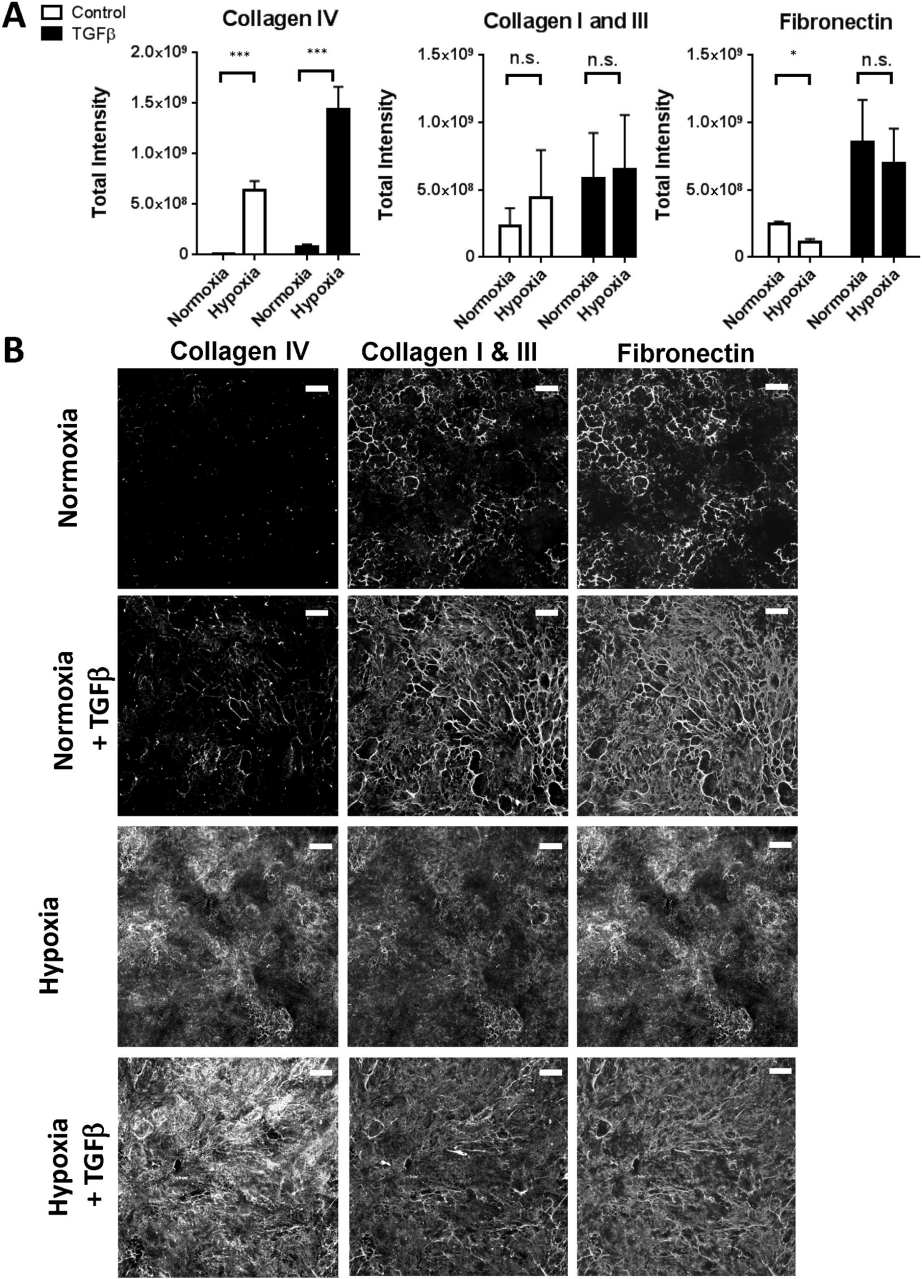
**Response of RPTECs to hypoxia.** (A) Epithelial cells grown for six days in either normoxic or hypoxic (2.5% O_2_) conditions with or without stimulation with 10 ng/mL TGF-β1 were assessed for extracellular matrix production using the immunofluorescence assay. Graphs shows data from three independent experiments, error bars indicate mean±s.e.m. ***P<0.001, *P<0.1, paired *t*-test performed on means from independent experiments. (B) Representative images for deposited extracellular matrix components from epithelial cells in A. Scale bars: 100 µm.

Hypoxia is another stimulus that can induce fibrosis *in vivo* through HIF-1α (Higgins et al., 2007), and it also increases the expression of mRNA for extracellular matrix components (Norman et al., 2000; Orphanides et al., 1997). We therefore tested the effects of hypoxia on epithelial cell ECM deposition. Although we did not observe a consistent increase in the stable deposition of fibrillar collagen I and III or fibronectin under hypoxic conditions, we did observe a large increase in collagen IV deposition that could be further increased by TGF-β1 addition (Fig. 5). Qualitatively, this matrix appeared to have a different structure. This is consistent with previous reports describing the sensitivity of these cells to hypoxia (Norman et al., 2000; Orphanides et al., 1997) and demonstrating that additional mature ECM is deposited which could contribute to further tubule dysfunction. These results show that in isolated culture, the accumulated ECM response of epithelial cells, whilst relatively insensitive to chemical agents, could be significantly modulated by hypoxia.

**Fig. 6.**
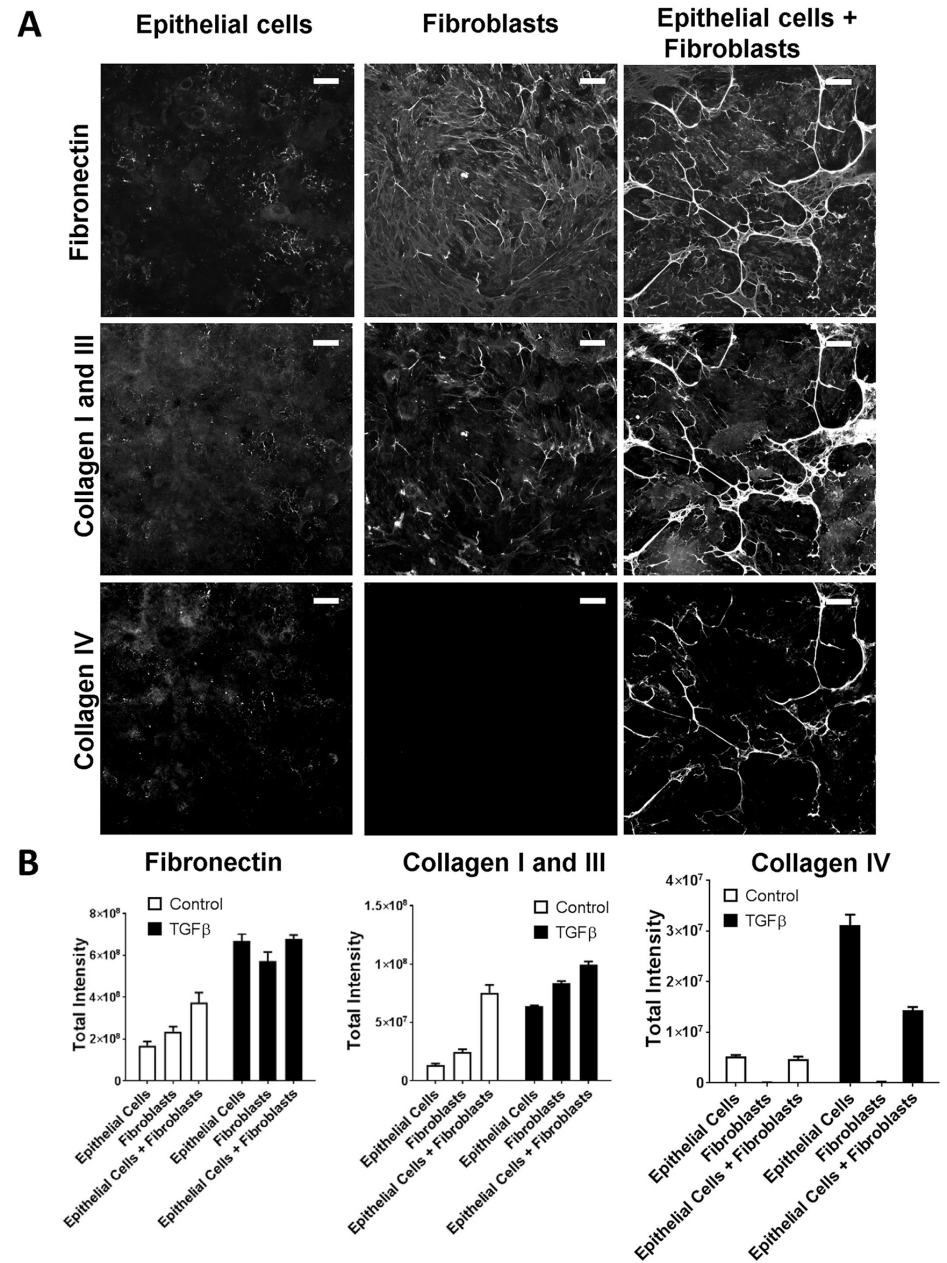
**Co-culture of fibroblasts and epithelial cells modulates extracellular matrix production.** (A) Representative images of the matrix accumulated by epithelial cells and fibroblasts in monoculture, or co-culture at a 1:1 cell ratio (with equal total numbers of cells per well) when cultured for six days without stimulation. Cells have been stripped, matrix-fixed and immunostained for matrix components: fibronectin (green), collagen I and III (red), collagen IV (blue). Scale bars: 100 µm. (B) Quantification of immunofluorescence for extracellular matrix components for cells as in A with and without stimulation with 10 ng/ml TGFβ1. Graphs show mean of four replicate wells ±s.e.m., one representative experiment of three shown.

**Fig. 7.**
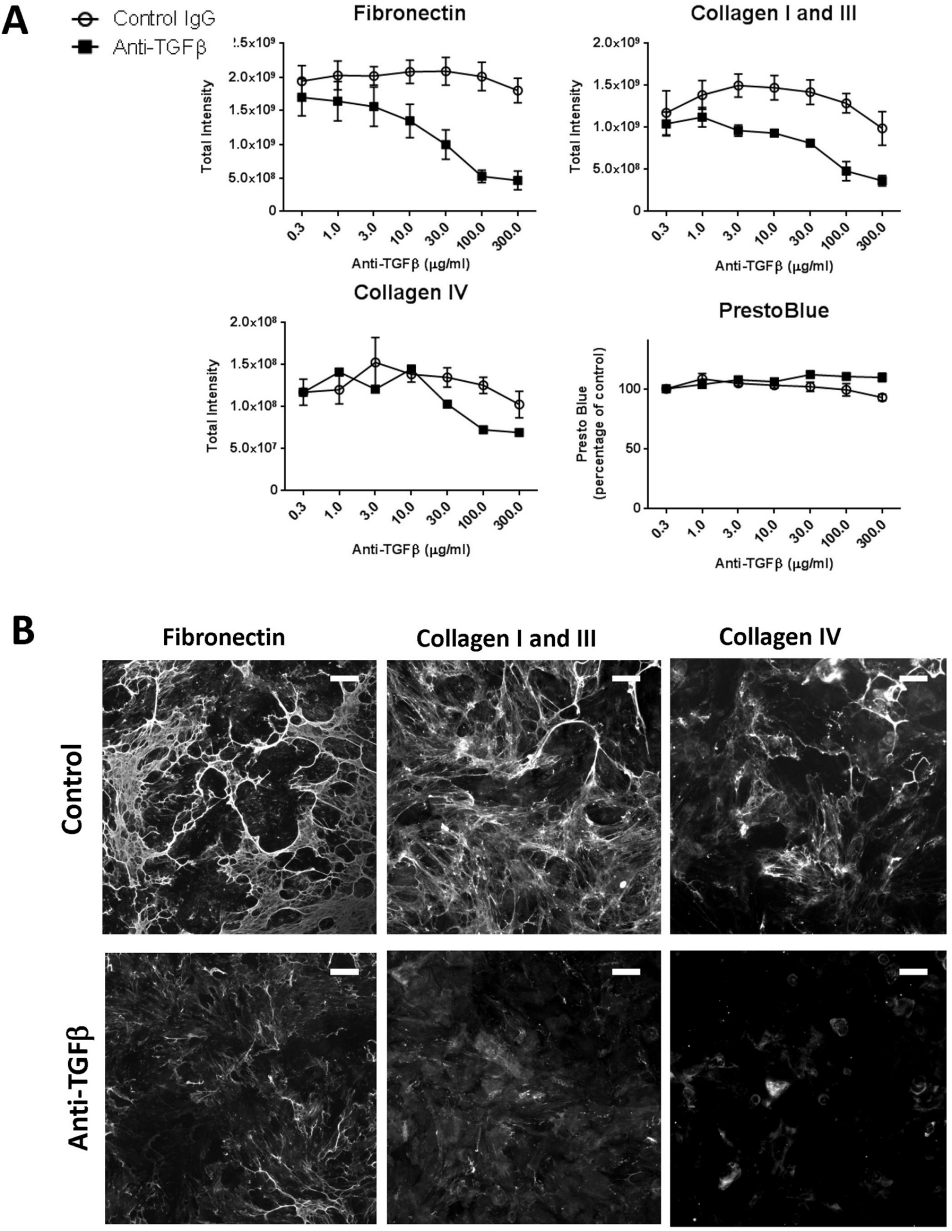
**TGFβ blockade reduces matrix deposition of co-cultured epithelial cells and fibroblasts.** (A) Epithelial cells and fibroblasts were co-cultured without additional stimulation for six days in the presence of the indicated concentrations of either a control or an anti-TGFβ antibody. Extracellular matrix was stained and analysed by the immunofluorescence method as previously described. Graphs show mean of three independent experiments ±s.e.m. (B) Panels show representative images of cells from A. Scale bars: 100 µm.

As a result of the relative unresponsiveness of epithelial cells to acute stress stimuli, we tested whether other cell types could alter epithelial cell extracellular matrix production. Although fibroblasts have a well-established role as producers of extracellular matrix, they express several factors that could alter tubular epithelial cells behaviour (Johnson et al., 1997). We co-cultured equal numbers of fibroblasts and epithelial cells and measured ECM by immunofluorescence. In the absence of exogenous stimulus, the co-culture of fibroblasts and epithelial cells resulted in higher levels of matrix deposition than could be accounted for by the same total cell number of either cell type alone and to a similar level as when stimulated by TGF-β1 (Fig. 6A,B). This increase was most marked for collagen I and III where the increase was over threefold greater than could be accounted for by either a culture comprising solely fibroblasts, or, the expected level from a 50:50 culture of the two cell types. This suggested that these components and their fibrillar assembly were significantly affected by the interactions between cells. TGF-β is a major fibrotic stimulus that can be produced and activated by both cell types which we have previously shown to drive the stable deposition of fibrillar extracellular matrix. Addition of an anti-TGF-β antibody to an unstimulated co-culture of epithelial cells with fibroblasts resulted in over a fourfold reduction in matrix deposition of fibronectin and collagen I /III with little change in cell viability (Fig. 7A,B). These results highlight the role of TGF-β as a major driver of ECM deposition in this co-culture system.

In summary, we have developed an immunofluorescence-based method for quantifying the components of a mature, accumulated ECM using human cells. Importantly we have only assessed the mature assembled ECM by stripping the cellular component and only imaging the remaining ECM. This is key in that it represents not only an increase in transcription and translation (as demonstrated by previous studies), but also post translational modification, assembly of the ECM, as well as the homeostatic balance attained with change to ECM clearance systems such as matrix metalloproteinases (MMPs) and plasmin. In response to TGF-β1, RPTECs demonstrated a clear deposition of ECM components including the fibrillary collagens I and III. Furthermore we have examined in a head-to-head comparison the capacity of epithelial and fibroblast responses to the most recognised pro-fibrotic cytokine TGF-β1 and shown that both cells have an almost equal capacity to generate components of a mature, deposited ECM.

Previous studies have demonstrated a role for hypoxia in increasing ECM production from RPTECs and we confirm here that this is deposited as a mature, fibrillary extracellular matrix (Norman et al., 2000; Orphanides et al., 1997). In contrast to cytokine stimulation and hypoxia, we observed a relative insensitivity of a mono-culture of epithelial cells to stress stimuli, and this may represent a missing component such as immune cells in this *in vitro* assay system (Lupher and Gallatin, 2006). In this study we have not normalised to cell counts as ultimately the amount of matrix remaining was most important to the fibrotic process we were trying to model. We observed a modest decrease (10%) in epithelial cell counts and a modest increase of 20% in PrestoBlue signal, a marker of metabolic activity, so normalisation would not substantially alter the results (data not shown).

Finally we observed that co-culture of epithelial cells and fibroblasts resulted in a largely TGF-β-dependent increase in basal matrix production. It is possible that as a result of tubule injury, interactions between fibroblasts and epithelial cells could be enhanced as in other fibrotic diseases (Sakai and Tager, 2013; Gabison et al., 2009; Moll et al., 2013). This increase could be a result of enzymes such as LOXL2 or TG2 which could cross-link and stabilise the matrix (Fisher et al., 2009). Alternatively renal fibroblasts have been reported to express TGF-β (Grupp et al., 2001) which could be activated by integrins expressed on epithelial cells (Rabb et al., 1996; Hinz, 2015). Cross-talk between these cell types has been previously reported as rat tubular epithelial cells have been reported to influence fibroblast α-smooth muscle actin expression (Lewis and Norman, 1998). This is also similar to an interaction between podocytes and endothelial cells which altered the ECM (Byron et al., 2014). Nevertheless many questions remain unanswered and the mechanism of this fibroblast-epithelial cross-talk is under active investigation. Whilst the contribution of epithelial cell-derived extracellular matrix to fibrosis *in vivo* is less clear, in diabetic nephropathy, epithelial cells show up-regulation of the extracellular matrix components collagen III and IV (Razzaque et al., 1995), and basement membrane thickening is a hallmark of chronic kidney disease which often precedes the increase in fibroblasts in experimental models (Fig. S1) (Gilbert and Cooper, 1999). The epithelial cell, or the fibroblast-epithelial cell, cross-talk could therefore represent important therapeutic targets in fibrotic disease.

Therefore consistent with our hypothesis, we have demonstrated that following cytokine stimulation, primary human epithelial cells can respond by producing a stable, fibrillar extracellular matrix that is comparable to that generated by activated fibroblasts. This ability to deposit ECM per se, especially the fibrillar collagens, as well as the enhanced deposition under hypoxic conditions and contact co-culture with fibroblasts, could make a significant contribution to fibrotic disease.

## MATERIALS AND METHODS

### Cell lines and stimulations

Human primary renal proximal tubular epithelial cells were purchased from Innoprot (Derio, Spain) and American Type Culture Collection (ATCC, Manassas, USA) and were maintained in Renal Epithelial Cell Basal Medium (ATCC) supplemented with Renal Epithelial Cell Growth Kit Components (ATCC). Human primary renal fibroblasts were purchased from Innoprot and maintained in DMEM F12 (Invitrogen) containing 10% FCS and supplemented with 2 mM L-Glutamine. For experiments, epithelial cell medium was used for both cell types. Cells were grown in 100% humidity and 5% CO_2_ at 37°C and were used until Passage 6. For hypoxia experiments, cells were grown in 2.5%O_2_ and 5% CO_2_ using a Panasonic MCO-19 M incubator. For stimulation with cytokines, cells were incubated with TGFβ1 10 ng/ml (RnD Systems, Minneapolis, USA) added at the start of the culture. Aristolochic acid and H_2_O_2_ were from Sigma and were used at the indicated concentrations. For TGF-β blockade, cells were incubated with anti-TGF-β MAB1835 or appropriate isotype control (RnD Systems) for the duration of the culture period.

### Flow cytometry

Cells were detached using Accutase and resuspended in staining buffer (PBS+0.2% BSA+0.1% sodium azide) before labelling as described for immunofluorescence with AlexaFluor488-conjugated anti-cytokeratin 18 (LDK18, EBioscience, San Diego, USA) or FITC conjugated Thy1.1 (AF-9, Abcam). Cells were then washed and analysed using a BD FACSCanto. Histograms were prepared using FlowJo (www.flowjo.com).

### Extracellular matrix quantification by ^14^C incorporation

Cells were grown in medium containing either a ^14^C-labelled amino acid mixture (Hartmann Analytic, Braunschweig, Germany) to measure total extracellular matrix or ^14^C proline (Perkin Elmer, Waltham, USA) to measure collagens, using 0.75 µCi/ml of each radiolabel. Cells were grown in 96-well CytoStar-T plates (Perkin Elmer) for 6 days. Cells were removed with 0.25 M ammonium hydroxide in 50 mM Tris pH 7.4 (Fisher et al., 2009), washed with PBS, and deposition of radiolabelled protein measured using a TriLux 1450 Microbeta Scintillation Counter (Perkin Elmer).

### Immunofluorescence

For immunofluorescence, cells were plated into 384-well or 96-well dark walled imaging plates (BD Biosciences, UK) in epithelial cell medium. For co-culture experiments, cells were plated in epithelial cell medium with equal total cell numbers. Cells were plated at 70% confluence and reached full confluence three days into the experiment.

For cell stains, cells were grown for 6 days before fixation using 4% PFA (Life Technologies, Carlsbad, USA). Cells were permeabilised using 0.1% saponin (Life Technologies) in PBS before staining with anti-FSP1 (Cell Signalling Technologies, #13018), α-smooth muscle actin (Sigma) and Alexa Fluor488-conjugated cytokeratin 18 (EBioscience, San Diego, USA, clone LDK18). Cells were then washed and incubated with Alexa Fluor555 or 647 anti-species secondary antibodies and DAPI for nuclear staining (Life Technologies). For extracellular matrix stains, cells were first removed using 0.25 M ammonium hydroxide in 50 mM Tris pH 7.4 as previously described (Fisher et al., 2009). Remaining matrix was then fixed using 100% methanol at −20°C followed by staining with Alexa Fluor488-conjugated anti-fibronectin (EBioscience, clone FN-3, 1 in 250 dilution), anti-collagen I and III (Merck Millipore, Billerica, USA, AB745 and AB747, 1:100 dilution) and eFluor660-conjugated anti-collagen IV (EBioscience, clone 1042, 1:100 dilution). Anti-collagen I and III were detected using Alexa Fluor555-conjugated anti-rabbit secondary antibodies (1:500, Life Technologies).

### Image acquisition

Images were acquired using a Cellomics Arrayscan (Life Technologies) with a 20× Objective, ORCA-ER camera (for cells) or 10× Objective, X1 camera (for ECM) and appropriate excitation and emission filter sets. Four images were acquired per well, with at least three replicate wells obtained in an experiment. For analysis of extracellular matrix fibrils we used the ‘Cell Health Profiling’ algorithm of the Cellomics Scan Software (Thermo Fisher Scientific, Waltham, USA) and used the entire image as the object. Staining above a fixed threshold was analysed and we measured the total intensity of the target. For analysis of nuclear area/counts, nuclei were detected as objects and the software reported counts and area data.

### RNA preparation and qRT-PCR

Cells were cultured for 48 h in 6-well plates, in the presence or absence of 10 ng/ml TGFβ1. Medium was removed and the cell monolayer washed once with cold PBS. Total RNA was isolated using RNEasy Plus Mini kit (Qiagen, Venlo, Netherlands), in accordance with the manufacturer's instructions. The resultant RNA was quantitated by spectrophotometry before being reverse transcribed using SuperScript VILO cDNA synthesis kit (Life Technologies) in accordance with the manufacturer's instructions. TaqMan PCR was performed in triplicate wells using TaqMan Gene Expression Master Mix (Life Technologies) with 1 μl cDNA/well. No Reverse Transcriptase (RT) and no template control wells were included in each experiment. The ΔΔCt method was used to normalise expression against the geometric mean of the housekeeper genes *B2M*, *HMBS* and *TBP* (Table 1).

**Table 1.**
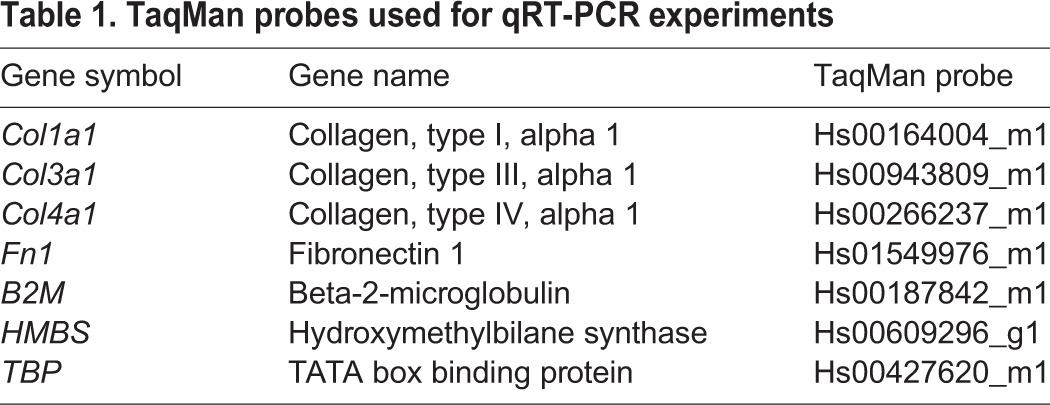
**TaqMan probes used for qRT-PCR experiments**

### Cell viability

Cell viability was measured using the Prestoblue reagent (Life Technologies) according to the manufacturer's instructions.

### Data analysis and statistics

Immunofluorescence and radioactivity data analysis to generate *P* values was performed using a linear mixed effect model on a log10 scale to help satisfy assumptions of equal variance (between the groups) and normality. Analyses were performed using SASv9.4 (SAS Institute). mRNA data analysis was performed using one-way ANOVA and GraphPad Prism software (GraphPad Software).
